# Identifying Immune Cell Infiltration and Effective Diagnostic Biomarkers in Lung Adenocarcinoma by Comprehensive Bioinformatics Analysis and *In Vitro* Study

**DOI:** 10.3389/fonc.2022.916947

**Published:** 2022-05-30

**Authors:** Xinyan Li, Xi Chen, Yixiao Yuan, RuiQing Zhai, William C. Cho, Xiulin Jiang

**Affiliations:** ^1^ Department of Pharmacy, Putuo Hospital, Shanghai University of Traditional Chinese Medicine, Shanghai, China; ^2^ Department of Neurosurgery, The Second Affiliated Hospital of Kunming Medical University, Kunming, China; ^3^ Department of Thoracic Surgery, The Third Affiliated Hospital of Kunming Medical University, Kunming, China; ^4^ College of Bioinformatics Science and Technology, Harbin Medical University, Harbin, China; ^5^ Department of Clinical Oncology, Queen Elizabeth Hospital, Hong Kong, Hong Kong SAR, China; ^6^ Key Laboratory of Animal Models and Human Disease Mechanisms of Chinese Academy of Sciences and Yunnan Province, Kunming Institute of Zoology, Kunming, China; ^7^ Kunming College of Life Science, University of Chinese Academy of Sciences, Beijing, China

**Keywords:** FAM72B, lung adenocarcinoma, prognosis biomarker, immune infiltration, ceRNA

## Abstract

Family with sequence similarity 72B (FAM72B) has been characterized in the regulation of neuronal development. Nevertheless, the prognostic value of FAM72B expression and its function in the immune microenvironment of lung adenocarcinoma (LUAD) currently remains elusive. In this study, by adopting bioinformatics methodology and experimental verification, we found that FAM72B was upregulated in lung cancer tissues and cell lines, and a higher FAM72B level predicted an unfavorable clinical outcome in LUAD patients. The knockdown of FAM72B significantly inhibited cell proliferation, cell migration, and induced cell apoptosis in LUAD. The receiver operating characteristic curve suggested that FAM72B had a high predictive accuracy for the outcomes of LUAD. Kyoto Encyclopedia of Genes and Genomes and Gene Set Enrichment Analyses confirmed that FAM72B-related genes were involved in cell proliferation and immune-response signaling pathway. Moreover, upregulated FAM72B expression was significantly associated with immune cell infiltration in the LUAD tumor microenvironment. Meanwhile, a potential ceRNA network was constructed to identify the lncRNA-AL360270.2/TMPO-AS1/AC125807.2/has-let-7a/7b/7c/7e/7f/FAM72B regulatory axis that regulates FAM72B overexpression in LUAD and is associated with a poor prognosis. We also confirmed that AL360270.2, TMPO-AS1, and AC125807.2 were significantly upregulated in LUAD cell lines than in human bronchial epithelial cells. In conclusion, FAM72B may serve as a novel biomarker in predicting the clinical prognosis and immune status for lung adenocarcinoma.

## Introduction

Lung cancer is the leading cause of cancer-related deaths worldwide, according to Cancer Statistics 2020. The incidence rate of lung cancer ranks second, while the death rate of lung cancer ranks first (1). Lung cancer includes small cell lung carcinoma (SCLC) and non-small cell lung carcinoma (NSCLC). NSCLC includes lung adenocarcinoma (LUAD), lung squamous cell carcinoma (LUSC), and large-cell lung carcinoma. The NSCLC cancer accounts for approximately 85% of all cases (2). Although the treatment of LUAD has improved, for the new LUAD pathogenesis, noninvasive diagnostic biomarkers with high sensitivity and specificity are still needed. Therefore, the discovery of potential key prognostic markers with more characteristics and value will help in the early prediction and treatment of LUAD at the molecular level.

Preliminary studies uncover that family with sequence similarity 72B (FAM72B) was upregulated in the nervous system, neuroblastoma, and breast adenocarcinoma (3). FAM72D was reported as a specific proliferation marker in myelomas (4). FAM72 (A–D) was increased during non-small cell and cancer cell proliferation and is present in the G2/M phase of the cell cycle (5). It has been confirmed that the depletion of FAM72A inhibited NSC cell proliferation and promotes cell differentiation (6). However, the prognostic value, diagnostic value, underlying function, and mechanisms of FAM72B in LUAD progression remain unclear.

Therefore, the aim of this study was to determine the effect of FAM72B on the progression of LUAD. In this study, we used The Cancer Genome Atlas (TCGA), Genotype–Tissue Expression (GTEx), and Kaplan–Meier plotter web to examine FAM72B expression and its correlation with prognosis. Furthermore, the association between FAM72B expression and immune infiltration was determined by TIMER database and single-sample Gene Set Enrichment Analyses (ssGSEA) method. The FAM72B–miRNA–lncRNA network was constructed by starBase. Finally, immunohistochemistry (IHC), qPCR, growth curve, transwell assay, wound healing, and cell flow cytometry experiments were performed to examine the biological function of FAM72B in LUAD cell lines. This study may provide evidence for prognostic biomarkers and therapeutic targets for LUAD.

## Materials and Methods

### TCGA Datasets

We acquired the gene profiles and clinical survival data of the LUAD samples from TCGA database (https://portal.gdc.cancer.gov/) (7). We utilized these data analyses of the correlation between FAM72B expression and relevant clinical information, including pathological stage and TNM stage. Because the sequencing data of normal tissues included in the TCGA are very limited and many patients lack transcriptome sequencing results for their normal tissues, we obtained data for normal tissues from the GTEx database. The above-mentioned analyses were constructed using the R (v4.0.3) software package ggplot2 (v3.3.3). R software v4.0.3 and ggplot2 (v3.3.3) were used for visualization. R software v4.0.3 was used for statistical analysis.

### LinkedOmics Database

LinkedOmics (http://www.linkedomics.org/login.php) is a publicly available portal that includes multi-omics data from all 32 TCGA cancer types and 10 Clinical Proteomics Tumor Analysis Consortium cancer cohorts. In this study, LinkedOmics was employed to obtain the genes that were significantly positively correlated with FAM72B expression in TCGA-LUAD.

### Kyoto Encyclopedia of Genes and Genomes and Gene Set Enrichment Analysis

The Kyoto Encyclopedia of Genes and Genomes (KEGG) pathways and related gene information were acquired from Gene Set Enrichment Analyses (GSEA) database. GSEA were conducted to examine the biological and molecular functions of FAM72B across different cancer types using a total of 300 genes that were positively correlated with FAM72B. All three analyses were performed using the R package Cluster Profiler. GSEA was also used to estimate the enrichment of various biological processes in each sample.

### Generation of Prognostic Risk Prediction Model

Univariate and multivariate Cox regression analyses were performed by applying the R3.6.1 package (version 2.41-1); then, the independent prognostic clinical factors of LUAD samples in the TCGA datasets were acquired with the *P <*0.05. Based on the independent prognostic factors screened in the previous step and the risk information discriminated by the prognostic prediction model, the 1-, 3-, and 5-year prognostic risk prediction models of the nomogram were built by applying R3.6.1 “rms”. In this research, Kaplan–Meier method was utilized to examine the prognostic values of FAM72B, miRNA and lncRNA expression—employing R packages of survminer—and survival.

### Immune Infiltration Analysis by TIMER Database and ssGSEA

The TIMER web server is a comprehensive resource for the systematical analysis of immune infiltrates across diverse cancer types (8). In this study, we employed the TIMER database to determine the association between FAM72B expression and the immune infiltrates (B cells, CD4+ T cells, CD8+ T cells, neutrophils, macrophages, and dendritic cells). We also utilized ssGSEA to examine the correlation between FAM72B expression and the LUAD immune infiltration of 24 tumor-infiltrating immune cells in tumor samples (9).

### Starbase Database

starBase v2.0 (http://starbase.sysu.edu.cn/) is a database which includes the RNA–RNA and protein–RNA interaction networks from CLIP-Seqdata sets generated by 37 independent studies (10). In this finding, starBase was used to predict the potential non-coding RNAs of FAM72B and determine the correlation between miRNAs and FAM72B in LUAD. Furthermore, Pearson’s correlation analysis was used to determine the relationship between lncRNAs and FAM72B expression in TCGA-LUAD.

### Cell Culture and Transfection

The BEAS-2B cell line was purchased from the Chinese Academy of Sciences Cell Bank (CASCB, China) and cultured in Bronchial Epithelial Cell Growth Medium (Lonza, CC-3170). The lung cancer cell lines, including HCC827, H1650, A549, and H1975, were purchased from the CASCB (China) with STR documents and were cultured in RPMI-1640 medium (Corning) supplemented with 10% fetal bovine serum (FBS) and 1% penicillin/streptomycin.

### Cell Migration Assay

Cell migration and invasion assays were conducted to explore the biological function of FAM72B on LUAD cells. For the transwell migration assay, 2.5 × 10^4^ cells/well in 100 μl serum-free medium were plated in a 24-well plate chamber insert, and the lower chamber was filled with 10% FBS. After incubation for 24 h, the cells were fixed with 4% paraformaldehyde, washed, and then stained with 0.5% crystal violet for further pictures to be captured.

### CCK8 Assay

We seeded the cells in 96-well plates at 2.5 × 10^3^ per well in 100 μl of complete medium and 10 μl of CCK-8 reagent (RiboBio, Guangzhou, China) for 1 h each day after 3 days of culture. We then used a microplate to measure the absorbance of each well at 450 nm. Each sample was evaluated in triplicate.

### Immunohistochemical Staining

For immunohistochemical staining, the sections were deparaffinized in xylene and rehydrated through graded ethanol. Antigen retrieval was performed for 20 min at 95°C with sodium citrate buffer (pH 6.0). After quenching the endogenous peroxidase activity with 3% H_2_O_2_ and blocking the non-specific binding with 1% bovine serum albumin buffer, the sections were incubated overnight at 4°C with the indicated primary antibodies. Following several washes, the sections were treated with horseradish peroxidase-conjugated secondary antibody for 40 min at room temperature and stained with 3,3-diaminobenzidine tetrahydrochloride. The slides were photographed with a microscope (Olympus BX43F, Japan). The photographs were analyzed based on the ratio of the staining with the Image-Pro Plus 7.0 software (Media Cybernetics, Inc., Silver Spring, MD, USA).

### Statistical Analyses

All statistical analyses were performed using R software, and receiver operating characteristic (ROC) curves were used to detect FAM72B cutoff values using pROC packages. For the data regarding the function of FAM72B, Graph Pad Prism 7.0 was used for statistical analyses.

## Results

### Expression Pattern of FAM72B in Human Cancers

**Figure 1:**
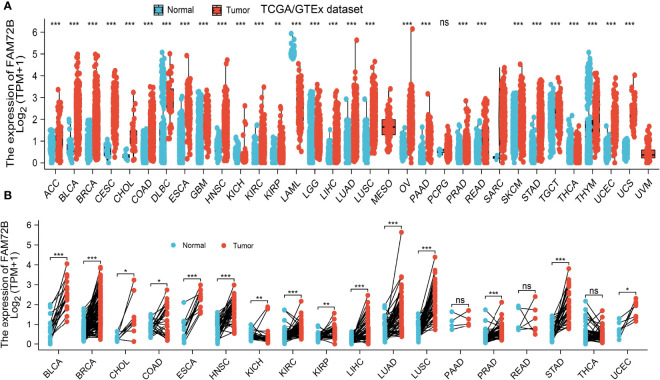
Expression level of FAM72B in pan-cancer. **(A)** The Cancer Genome Atlas (TCGA) cohort determined the expression of FAM72B in pan-cancer. **(B)** TCGA cohort determined the expression of FAM72B in paired cancer tissues and adjacent normal tissues. ACC, adrenocortical carcinoma; BLCA, bladder urothelial carcinoma; BRCA, breast invasive carcinoma; CESC, cervical squamous cell carcinoma and endocervical adenocarcinoma; CHOL, cholangiocarcinoma; COAD, colon adenocarcinoma; DLBC, lymphoid neoplasm diffuse large B-cell lymphoma; ESCA, esophageal carcinoma; GBM, glioblastoma multiforme; HNSC, head and neck squamous cell carcinoma; KICH, kidney: chromophobe; KIRC, kidney renal clear cell carcinoma; KIRP, kidney renal papillary cell carcinoma; LAML, acute myeloid leukemia; LUAD, brain lower-grade glioma; LIHC, liver hepatocellular carcinoma; LUAD, lung adenocarcinoma; LUSC, lung squamous cell carcinoma; MESO, mesothelioma; OV, ovarian serous cystadenocarcinoma; PAAD, pancreatic adenocarcinoma; PCPG, pheochromocytoma and paraganglioma; PRAD, prostate adenocarcinoma; READ, rectum adenocarcinoma; SARC, sarcoma; SKCM, skin cutaneous melanoma; STAD, stomach adenocarcinoma; TGCT, testicular germ cell tumors; THCA, thyroid carcinoma; THYM, thymoma; UCEC, uterine corpus endometrial carcinoma; UCS, uterine carcinosarcoma; UVM, uveal melanoma. NS, *P* > 0.05, *P < 0.05, **P < 0.01, ***P < 0.001.

To determine the mRNA expression pattern of FAM72B in diverse cancer types, we used TCGA and GTEx datasets in conducting an analysis. The results indicated that FAM72B was highly expressed in 25 of the 33 cancers compared with normal tissue (**Figure 1A**). We further determine FAM72B expression in paired cancer tissues and adjacent normal tissues by utilizing the TCGA datasets. We found that FAM72B expression was significantly higher in 15 of the 18 cancers compared with normal tissue (**Figure 1B**). These results show that FAM72B was highly expressed in various human cancers.

### FAM72B Was Upregulated in Lung Adenocarcinoma

**Figure 2:**
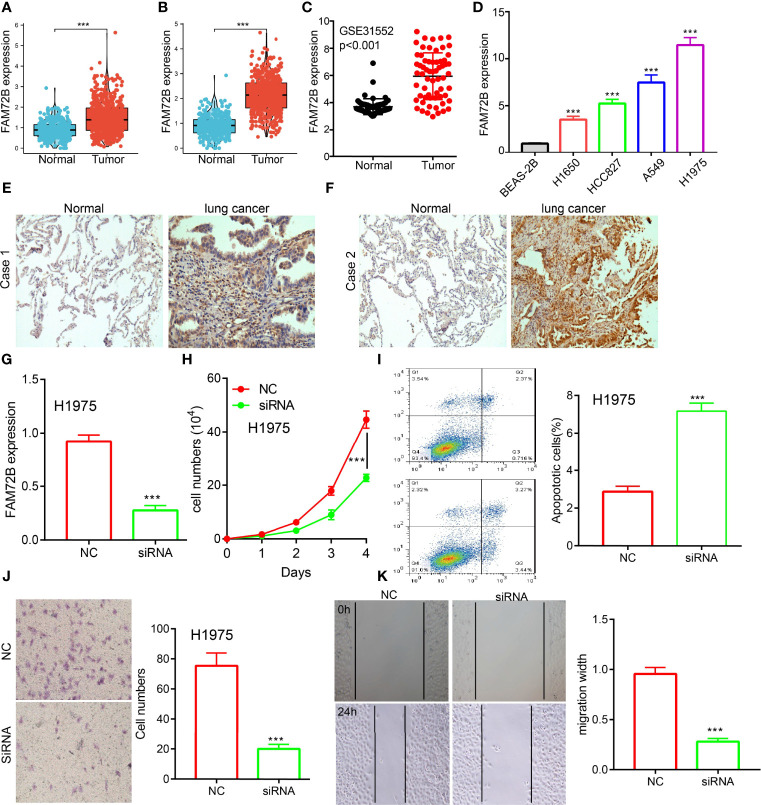
Knockdown of FAM72B inhibited lung adenocarcinoma (LUAD) progression. **(A, B)** FAM72B was overexpressed in LUAD and lung squamous cell carcinoma as examined by The Cancer Genome Atlas. **(C)** Relative FAM72B expression as examined by the Gene Expression Omnibus dataset. **(D)** mRNA level of FAM72B in the lung cancer cell lines compared to normal human bronchial epithelial cell line: BEAS-2B determined by qPCR assay. **(E, F)** Protein expression of FAM72B in lung cancer tissues as examined by immunohistochemical staining assay. **(G)** Establishment of FAM72B knockdown cell lines in H1975 verified by real-time RT-PCR. **(H)** Knockdown of FAM72B significantly inhibits cell proliferation as determined by CCK8 assay. **(I)** Knockdown of FAM72B significantly induced cell apoptosis. **(J, K)** knockdown of FAM72B inhibited LUAD cell migration. NC, negative control; siRNA, FAM72B siRNA. ***P < 0.001.

To examine the FAM72B expression level in LUAD, we analyzed FAM72B expression based on the TCGA and Human Protein Atlas database. We found that FAM72B was upregulated both in LUAD and LUSC than in normal tissues (**Figures 2A, B**). Consistent with the above-mentioned results, the Gene Expression Omnibus (GEO) dataset also demonstrated that the FAM72B mRNA level was obviously increased in lung cancer tissues (**Figure 2C**). Furthermore, we showed that the RNA of FAM72B was upregulated in LUAD cells lines, especially in H1975 cells (**Figure 2D**). Finally, to prove the above-mentioned findings, immunohistochemical staining assay was conducted to examine the protein of FAM72B in lung cancer tissues. The results confirmed the upregulation in lung cancer tissues than in normal lung tissues (**Figures 2E, F**).

Given that the biological function of FAM72B in LUAD remains unclear, we further determine the potential function of FAM72B on LUAD cell proliferation and migration. The qRT-PCR assay showed that the expression of FAM72B mRNA was significantly decreased in H1975 cells after treatment with the targeted siRNA (**Figure 2G**). The growth curve assays demonstrated that FAM72B depletion significantly inhibits the cell proliferation ability of LUAD (**Figure 2H**). Moreover, we show that the knockdown of FAM72B promotes cell apoptosis (**Figure 2I**). Furthermore, to validate whether FAM72B is critical for cell migration, we performed transwell and wound healing assays and revealed that FAM72B knock-down significantly inhibited the cell proliferation ability of LUAD (**Figures 2J, K**).

### FAM72B Expression and Clinico-pathological Characteristics of Lung Adenocarcinoma

**Figure 3:**
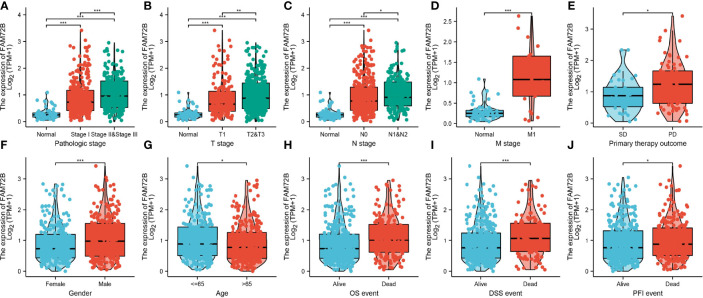
Relationship between FAM72B mRNA expression and clinico-pathological parameters in patients with lung adenocarcinoma. **(A–J)** Correlation between FAM72B expression and the clinical parameters, including the pathological stage, TNM stage, primary therapy outcome, gender, age, overall survival event, disease-specific survival event, and progression-free event. *P < 0.05, **P < 0.01, and ***P < 0.001.

We also assessed the correlation between FAM72B expression and the clinicopathological characteristics of LUAD samples. As shown in **Figures 3A–J**, FAM72B expression was significantly associated with pathological stage, TNM stage, primary therapy outcome, gender, age, OS, DSS, and progression-free survival (PFS) in LUAD (**Figures 3H–J**). The logistic regression analysis also suggested that increased FAM72B expression was associated with T stage (T2 and T3 and T4 *vs*. T1; *P* < 0.001), N stage (N1 and N2 and N3 *vs*. N0; *P* = 0.040), pathologic stage (stage III and stage IV *vs*. stage I and stage II; *P* = 0.025), and gender (male *vs*. female) (*P* < 0.001) (**Table 1**).

**Table 1:** Logistic regression analyzed the correlation between FAM72B expression and the clinical pathological characteristics in lung adenocarcinoma.

Characteristics	Total (*N*)	Odds ratio	*P*-value
T stage (T2 and T3 and T4 *vs*. T1)	532	1.847 (1.282–2.674)	0.001
N stage (N1 and N2 and N3 *vs*. N0)	519	1.472 (1.019–2.133)	0.040
M stage (M1 *vs*. M0)	386	2.044 (0.885–5.120)	0.105
Pathologic stage (stage III and stage IV *vs*. stage I and stage II)	527	1.628 (1.065–2.507)	0.025
Gender (male *vs*. female)	535	1.905 (1.352–2.693)	<0.001
Age (>65 *vs*. ≤65)	516	0.711 (0.502–1.004)	0.053
Smoker (yes *vs*. no)	521	1.626 (0.993–2.697)	0.056

### Analysis of the Diagnostic and Prognostic Value of FAM72B in LUAD

**Figure 4:**
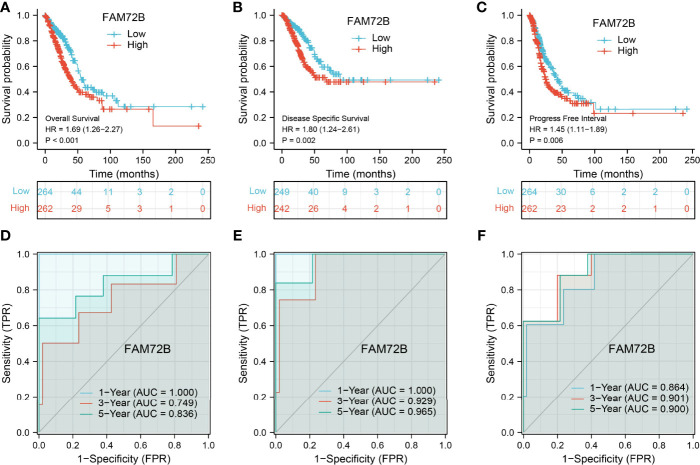
Prognostic and diagnostic values of FAM72B in lung adenocarcinoma (LUAD). **(A–C)** Kaplan–Meier survival curves suggested that LUAD patients with a higher FAM72B expression exhibited poor overall survival, disease-specific survival, and progression-free survival as determined by The Cancer Genome Atlas-LUAD dataset. **(D–F)** Receiver operating characteristic curves were used to determine the diagnostic value of FAM72B in lung adenocarcinoma.

**Figure 5:**
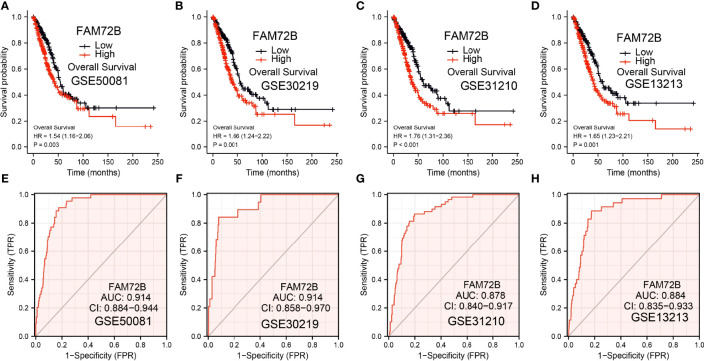
Validation of the prognostic and diagnostic values of FAM72B in lung adenocarcinoma (LUAD). **(A–D)** Validation of the prognosis of FAM72B in LUAD using the Gene Expression Omnibus (GEO) dataset. **(E–H)** Validation of the diagnostic values of FAM72B in LUAD using the GEO dataset.

The relationship between FAM72B expression and OS, DSS, and PFS in LUAD patients was examined by a Kaplan–Meier curve. We found that increased FAM72B expression was correlated with poor OS, DSS, and PFS in patients with LUAD (**Figures 4A–C**). According to time-dependent ROC, the FAM72B expression level had a relatively good performance in predicting the 1-year (C statistics, 1.0), 3-year (C statistics, 0.749), and 5-year overall survival (C statistics, 0.8363) in LUAD patients (**Figure 4D**), had a better performance in predicting the 1-year (C statistics, 1.00), 3-year (C statistics, 0.929), and 5-year disease-free survival (C statistics, 0.965) in LUAD patients (**Figure 4E**), and had a relatively good performance in predicting the 1-year (C statistics, 0.864), 3-year (C statistics, 0.901), and 5-year progression-free survival (C statistics, 0.900) in LUAD patients (**Figure 4F**). We also utilized the GEO dataset to validate the above-mentioned results. We showed that the upregulation of FAM72B expression was related to adverse clinical outcomes in patients with lung cancer (**Figures 5A–D**). We further explore the diagnostic significance of FAM72B in lung cancer; a ROC curve analysis was performed. The ROC curve analysis confirmed that the area under the ROC curve values of FAM72B were 0.914, 0.914, 0.878, and 0.884 in various GEO datasets, respectively (**Figures 5E–H**). These results confirmed that FAM72B may be a promising biomarker for differentiating LUAD.

### Validation of the Prognostic Value of FAM72B Based on Various Subgroups

**Figure 6:**
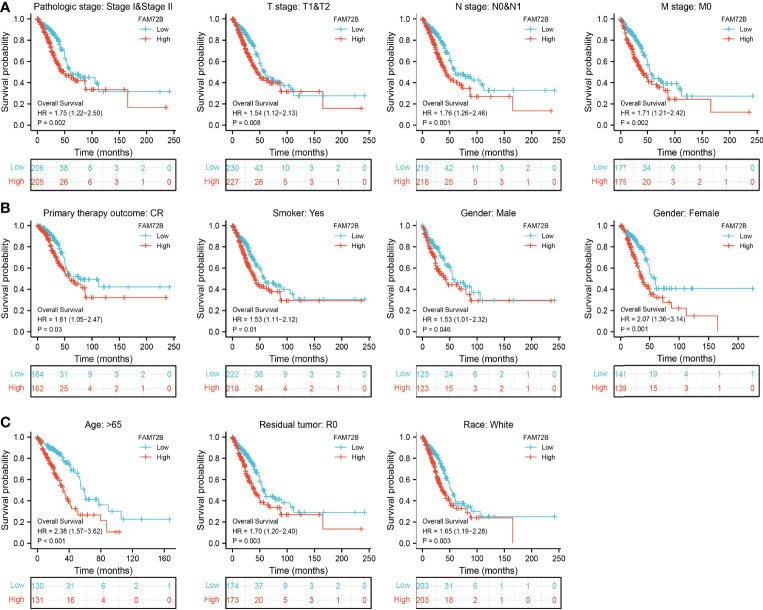
Overall survival of FAM72B based on diverse subgroups. **(A–C)** Correlations between the FAM72B expression level and the overall survival in different clinical subgroups of lung adenocarcinoma in The Cancer Genome Atlas database, including stages I and II, T1 and T2, N0 and N1, M0, CR, smoker, gender, age >65, residual tumor, and white.

We further determine the prognostic values of FAM72B in various clinical subgroups, including the pathological stage, TNM stage, gender, primary therapy outcome, age, residual tumors, race, and smoker. The results suggested that the upregulated FAM72B level is associated with a poor clinical outcome in patients with lung cancer (**Figures 6A–C**).

### Univariate and Multivariate Cox Regression Analyses of Different Parameters on Overall Survival

**Figure 7:**
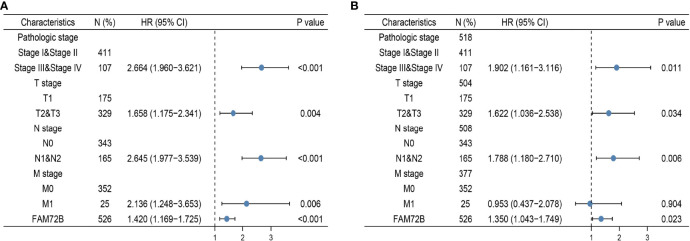
Forest plot of univariate and multivariate Cox regression analyses in lung adenocarcinoma **(A, B)**.

We performed univariate Cox regression analysis in the TCGA-LUAD cohort to determine whether the FAM72B expression level and the pathologic stage might be valuable prognostic biomarkers. The univariate COX analysis suggested that a higher expression of FAM72B, pathologic stage, and TNM stage, respectively, were correlated with a poor clinical outcome in LUAD patients. To ascertain whether the FAM72B expression level could be an independent prognostic factor for patients with LUAD, multivariate Cox regression analysis was performed. The multivariate COX analysis shows that a higher FAM72B expression, as well as pathologic stage and TNM stage, was a significant independent prognostic factor in the TCGA-LUAD cohort that directly correlated with poor overall survival (**Figures 7A, B**).

### Construction and Validation of FAM72B-Based Nomogram

**Figure 8:**
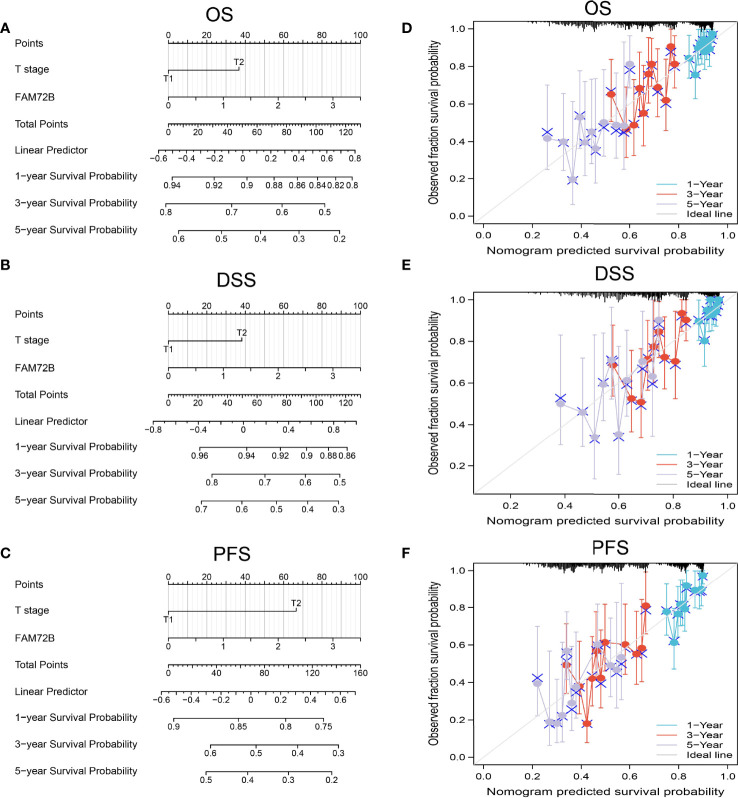
Nomogram and calibration curve for predicting the probability of 1-, 3-, and 5-year overall survival, disease-specific survival, and progression-free survival for lung adenocarcinoma (LUAD) patients. **(A–C)** A nomogram integrates FAM72B and other prognostic factors in LUAD from The Cancer Genome Atlas data. **(D–F)** Calibration curve of the nomogram.

The multivariate analysis result confirmed that FAM72B is an independent prognostic factor in LUAD. We then constructed a prediction model for overall survival and progression-free survival by integration of FAM72B expression. We established a nomogram to integrate FAM72B as a LUAD biomarker. Higher total points on the nomogram for OS, DSS, and PFS, respectively, indicated a worse prognosis (**Figures 8A–F**).

### KEGG Enrichment Analysis

**Figure 9:**
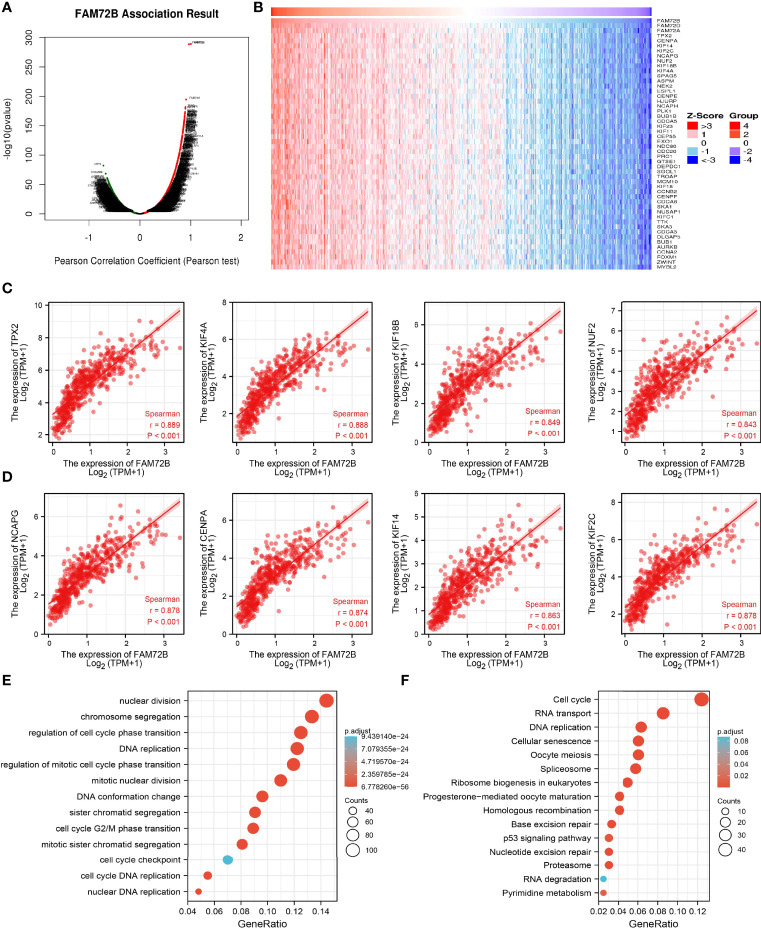
Kyoto Encyclopedia of Genes and Genomes (KEGG) enrichment analysis of FAM72B. **(A–D)** Genes that were significantly positively correlated with FAM72B expression in lung adenocarcinoma (LUAD) based on our The Cancer Genome Atlas-LUAD data. **(E, F)** Gene Ontology and KEGG enrichment analysis of FAM72B in LUAD.

To determine the potential function of FAM72B in LUAD progression, LinkedOmics database was utilized to obtain the top 100 genes that were significantly positively correlated with FAM72B expression (**Figures 9A, B**). The correlation analysis of FAM72B expression and the top 8 co-expressed genes in TCGA LUAD is shown in **Figures 9C, D**. For the terms of biological process, FAM72B is mainly involved in nuclear division, chromosome segregation, regulation of cell cycle phase transition, DNA replication, regulation of mitotic cell cycle phase transition, mitotic nuclear division, and cell cycle G2/M phase transition (**Figure 9E**). The KEGG enrichment analysis suggested that these genes participate in cell cycle, RNA transport, DNA replication, cellular senescence, spliceosome, and 53 signaling pathways (**Figure 9F**).

**Figure 10:**
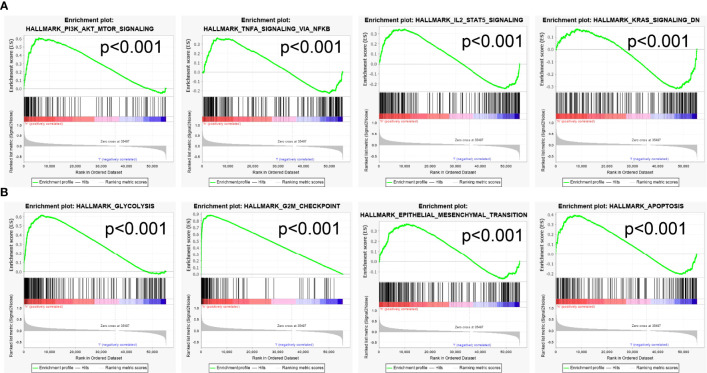
Gene Set Enrichment Analyses identification of FAM72B-related signaling pathways **(A, B)**.

To explore the possible mechanism of FAM72B in LUAD, the GSEA analysis was carried out on the different genes. The GSEA also showed that pathways, including the PI3K AKT MTOR signaling pathway, TNFA signaling pathway, IL2 STAT5 signaling pathway, KRAS signaling pathway, glycolysis, G2M checkpoint, epithelial-to-mesenchymal transition (EMT), and apoptosis, were significantly enriched in the high-FAM72B-expression group (**Figures 10A, B**).

### Correlation Between FAM72B Expression and Immune Infiltration

**Figure 11:**
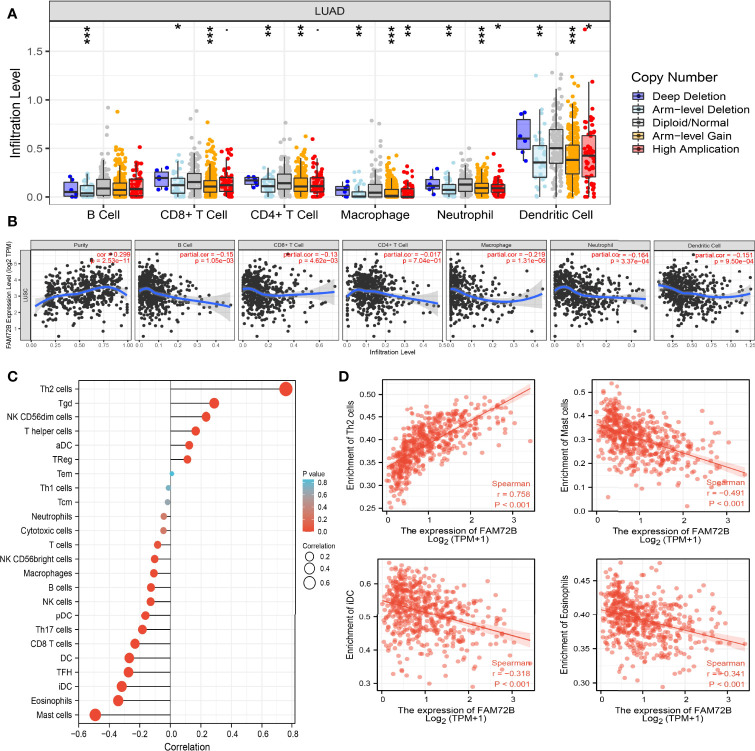
Correlation analysis of FAM72B expression and infiltration levels of immune cells in lung adenocarcinoma (LUAD). **(A)** Correlation between FAM72B CNV and infiltration levels of immune cells in LUAD. **(B)** FAM72B was significantly associated with tumor purity and negatively correlated with the infiltration of different immune cells using the TIMER database. **(C, D)** Correlation between FAM72B expression and the infiltration levels of 24 immune cells in LUAD by single-sample Gene Set Enrichment Analysis. *P < 0.05, **P < 0.01, ***P < 0.001.

Given that the GSEA indicated that FAM72B may be correlated with immune response regulation, we subsequently examined the relationship between FAM72B expression and immune cell infiltration. We found that the somatic copy number alterations of FAM72B significantly affect the infiltration level of B cells, CD4+ T cells, CD8+ T cells, neutrophils, macrophages, and dendritic cells in LUAD (**Figure 11A**). Furthermore, our results confirmed that most immune cells in the tumor microenvironment, including B cells, CD4+ T cells, CD8+ T cells, neutrophils, macrophages, and dendritic cells, were negatively associated with FAM72B expression in LUAD (**Figure 11B**).

Additionally, to validate the above-mentioned results, we employed the ssGSEA method to determine the association between FAM72B expression and 24 tumor-infiltrating lymphocytes in LUAD. The results suggested that FAM72B was positively associated with the infiltration of Th2 cells, Tgd, and NK CD56dim cells but negatively associated with the infiltration of mast cells, eosinophils, TFH, iDC, and DC in LUAD (**Figures 11C, D**).

### Prediction and Construction of FAM72B ceRNA Regulatory Network in LUAD

**Figure 12:**
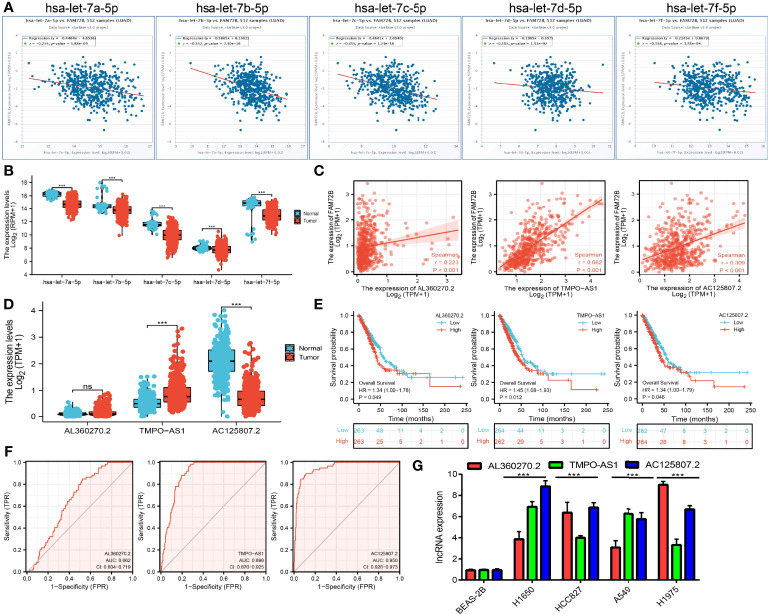
Analysis of the potential miRNAs and lncRNAs of FAM72B. **(A)** Correlations between FAM72B expression and miRNAs (has-let-7a/7b/7c/7e/7f-5p) in lung adenocarcinoma (LUAD). **(B)** Expression level of miRNAs (has-let-7a/7b/7c/7e/7f-5p) in LUAD. **(C)** Correlations between FAM72B expression and lncRNAs (AL360270.2, TMPO-AS1, and AC125807.21) in LUAD. **(D–F)** Expression levels and prognostic and diagnostic values of lncRNAs (AL360270.2, TMPO-AS1, and AC125807.21) in LUAD. **(G)** Expression of AL360270.2, TMPO-AS1, and AC125807.21 in LUAD cell lines by qPCR assay. NS, *P* > 0.05, ***P < 0.001.

To further determine the upstream potential molecular mechanism of FAM72B in LUAD, we therefore attempted to predict and construct the potential ceRNA regulatory network of FAM72B in LUAD. We utilized starBase to predict the potential miRNAs of FAM72B and identified 41 potential miRNAs (**Supplementary Table S1**). Furthermore, we analyzed the relationship between these miRNAs and the FAM72B expression levels and showed that has-let-7a-5p, has-let-7b-5p, has-let-7c-5p, has-let-7e-5p, and has-let-7f-5p were significantly negatively associated with FAM72B expression in LUAD (**Figure 12A**). A further analysis revealed that has-let-7a-5p, has-let-7b-5p, has-let-7c-5p, has-let-7e-5p, and has-let-7f-5p were downregulated in LUAD (**Figure 12B**). Therefore, we decided to select has-let-7a-5p, has-let-7b-5p, has-let-7c-5p, has-let-7e-5p, and has-let-7f-5p and conducted a downstream analysis. We further predicted, by using starBase tools, that the upstream lncRNAs might bind to has-let-7a-5p, has-let-7b-5p, has-let-7c-5p, has-let-7e-5p, and has-let-7f-5p. Based on the ceRNA hypothesis, miRNAs have an opposite co-expression correlation with mRNAs and lncRNAs, whereas lncRNAs have a positive co-expression correlation with mRNA (11). Based on starBase and Pearson’s correlation analysis, we found three lncRNAs, including AL360270.2, TMPO-AS1, and AC125807.2, to be negatively correlated with miR-125a-5p and positively correlated with FAM72B expression in LUAD, respectively (**Figure 12C**). We also show that AL360270.2, TMPO-AS1, and AC125807.2 were increased in LUAD, and a higher AL360270.2, TMPO-AS1, and AC125807.2 expression had a poor prognosis compared to the high-expression group (**Figures 12D, E**). The ROC curve analysis confirmed that AL360270.2, TMPO-AS1, and AC125807.2 may be promising biomarkers in LUAD (**Figure 12F**). Finally, we used qQT-PCR assay to detect the expression of AL360270.2, TMPO-AS1, and AC125807.2 in LUAD cell lines. The results suggested that AL360270.2, TMPO-AS1, and AC125807.2 were significantly upregulated in LUAD cell lines than in human bronchial epithelial cells (BEAS-2B) (**Figure 12G**).

## Discussion

LUAD is still the most afflicting cancer in the world, and the 5-year survival rate of lung cancer is only 10–15% in many countries (12). Previous studies have confirmed that FAM72B has been found to play a crucial role in maintaining the nervous system development (5). Nevertheless, there are few research studies on the synthesis study of FAM72B in LUAD. In this finding, we analyzed FAM72B expression, prognostic value, diagnostic values, ceRNA network, and correlation with tumor immune cell infiltration in LUAD for the first time.

In this project, we found a high level of FAM72B in various human cancers by analyzing the GTEX and TCGA cohorts. Moreover, we uncover that the mRNA and protein levels of FAM72B in the LUAD samples were remarkably higher than those in the normal control group through *in vitro* experiments and IHC staining, and the analysis results are the same as the above-mentioned studies. The elevated FAM72B expression was associated with an adverse pathological stage and TNM stage. The Kaplan–Meier curve analysis suggested that FAM72B expression was correlated with OS, disease-free survival, and PFS in the LUAD patients of the TCGA data. We also analyzed the potential of FAM72B expression to predict LUAD by conducting ROC curves and suggested that FAM72B has a high accuracy in predicting the outcomes of normal tissues and LUAD. Our findings are consistent with those of previous research. FAM72B was increased in GBM and correlated with poorer survival of patients (5).

The logistic regression analysis also suggested that increased FAM72B expression was associated with T stage (T2 and T3 and T4 *vs*. T1) (*P* < 0.001), N stage (N1 and N2 and N3 *vs*. N0) (*P* = 0.040), pathologic stage (stage III and stage IV *vs*. stage I and stage II) (*P* = 0.025), and gender (male *vs*. female) (*P* < 0.001). Next, the univariate and multivariate analysis results suggested that FAM72B expression was an independent factor associated with the survival of patients.

Given that FAM72B was highly expressed in LUAD tissues and cell lines, we also uncover that the knockdown of FAM72B significantly reduced the proliferation and migration abilities of LUAD cells. Cell apoptosis was found to play a crucial role in maintaining cell growth. In this study, we determine that the depletion of FAM72B significantly promotes cell apoptosis in LUAD.

Previous studies reported that FAM72B was upregulated in the nervous system, neuroblastoma, and breast adenocarcinoma (3). FAM72B was identified as a member of a 7-gene signature in prostate cancer and correlated with poor prognosis in patients with prostate cancer (13). It has been shown that FAM72B promotes NSC and cancer cell proliferation and is present in the G2/M phase of the cell cycle (6). Another study confirmed that the knockdown of FAM72B inhibited the cell proliferation of human fibroblasts (14). In this study, we investigated the underlying mechanisms through which FAM72B was involved in the progression of LUAD. The GSEA enrichment suggested that FAM72B was significantly associated with the PI3K AKT MTOR signaling pathway, TNFA signaling pathway, IL2 STAT5 signaling pathway, KRAS signaling pathway, glycolysis, G2M checkpoint, EMT, and apoptosis.

By the analysis of TIMER database, we discovered that FAM72B expression in LUAD was negatively associated with the expression levels of B cells, CD4+ T cells, CD8+ T cells, neutrophils, macrophages, and dendritic cells but positively associated with tumor purity. Moreover, FAM72B CNV was remarkably correlated with B cells, CD4+ T cells, CD8+ T cells, neutrophils, macrophages, and dendritic cells. These analyses point out that FAM72B may be participating in the immune response to the LUAD tumor microenvironment, particularly to B cells and CD4+ T cells.

It has been well documented that the ceRNA network plays an important role in the progression of lung cancer (15). The main finding of this study was the identification of a prognosis-related ceRNA regulatory network (lncRNA-AL360270.2, TMPO-AS1, and AC125807.2/has-let-7a/7b/7c/7e/7f-5p/FAM72B) in NSCLC. In the ceRNA regulatory network, has-let-7a/7b/7c/7e/7f-5p was significantly negatively correlated with FAM72B expression, and lncRNA-AL360270.2, TMPO-AS1, and AC125807.2 were significantly positively correlated with FAM72B expression. It has been confirmed that has-let-7a/7b/7c/7e/7f-5p inhibited LUAD progression *via* targeting BCL2L1 and IGF1R (16). We conducted correlation analysis, expression analysis, and prognosis analysis. has-let-7a/7b/7c/7e/7f-5p was selected as the most potential upstream-tumor-suppressive miRNA of FAM72B. Finally, we also constructed a FAM72B-related ceRNA network, which identified lncRNA-AL360270.2, TMPO-AS1, and the AC125807.2/has-let-7a/7b/7c/7e/7f-5p/FAM72B regulatory axis. In fact, lncRNA TMPO-AS1 was reported to promote lung adenocarcinoma progression and is negatively regulated by miR-383-5p (17). These results consistently suggest that lncRNA-AL360270.2, TMPO-AS1, and AC125807.2/has-let-7a/7b/7c/7e/7f-5p/FAM72B are a poor prognosis-associated ceRNA regulatory network in LUAD. In addition, the ceRNA network that we constructed could also elucidate the regulatory mechanism of FAM72B overexpression and poor prognosis in LUAD. However, although the ceRNA network of FAM72B was constructed by database analyses, more biological function assays are needed to further prove our analysis.

This study improves our understanding of the correlation between FAM72B and LUAD, but some limitations still exist. First, although we explored the correlation between FAM72B and immune infiltration in LUAD patients, there is a lack of experiments to validate the function of FAM72B in the tumor microenvironment regulation of LUAD. Second, we uncover that the knockdown of FAM72B inhibits the cell proliferation and cell migration of LUAD. However, the potential molecular mechanisms of FAM72B in cancer progression need to be explored in further studies.

## Conclusion

This finding demonstrated, for the first time, the clinical significance and biological function of FAM72B in lung adenocarcinoma. Therefore, the lncRNA-AL360270.2, TMPO-AS1, and AC125807.2/has-let-7a/7b/7c/7e/7f-5p/FAM72B regulatory network may serve as a novel prognostic biomarker and potential therapeutic target for LUAD treatment. In summary, FAM72B may serve as a promising diagnostic and prognostic biomarker for LUAD.

## Data Availability Statement

The datasets presented in this study can be found in online repositories. The names of the repository/repositories and accession number(s) can be found in the article/**Supplementary Material**.

## Author Contributions

XL, XC, and YY designed this work and performed the related assay. RZ analyzed the data. WC and XJ supervised the study and wrote the manuscript. All authors contributed to the article and approved the submitted version.

## Funding

This work was supported by the National Nature Science Foundation of China (82160512, 30960398, 81460174, and 81360126), the Yunnan Applied Basic Research Projects (2017FE467 and 2018FE001), the Applied Basic Research Project of Yunnan Provincial Science and Technology Department, Kunming Medical University (number 2020001AY070001-117), and the Open Project of The First People’s Hospital of Yunnan Province Clinical Medicine Center (2021LCZXXF‐XZ03).

## Conflict of Interest

The authors declare that the research was conducted in the absence of any commercial or financial relationships that could be construed as a potential conflict of interest.

## Publisher’s Note

All claims expressed in this article are solely those of the authors and do not necessarily represent those of their affiliated organizations, or those of the publisher, the editors and the reviewers. Any product that may be evaluated in this article, or claim that may be made by its manufacturer, is not guaranteed or endorsed by the publisher.

## References

[1] Siegel RL Miller KD Jemal A. Cancer Statistics, 2020. CA: A Cancer J Clin (2020) 70(1):7–30. doi: 10.3322/caac.21590 31912902

[2] Molina JR Yang P Cassivi SD Schild SE Adjei AA. Non-Small Cell Lung Cancer: Epidemiology, Risk Factors, Treatment, and Survivorship. Mayo Clin Proc (2008) 83(5):584–94. doi: 10.1016/S0025-6196(11)60735-0 PMC2718421 18452692

[3] Nehar S Mishra M Heese K. Identification and Characterisation of the Novel Amyloid-Beta Peptide-Induced Protein P17. FEBS Lett (2009) 583(19):3247–53. doi: 10.1016/j.febslet.2009.09.018 19755123

[4] Chatonnet F Pignarre A Sérandour AA Caron G Avner S Robert N. The Hydroxymethylome of Multiple Myeloma Identifies FAM72D as a 1q21 Marker Linked to Proliferation. Haematologica (2020) 105(3):774–83. doi: 10.3324/haematol.2019.222133 PMC7049362 31221779

[5] Rahane CS Kutzner A Heese K. A Cancer Tissue-Specific FAM72 Expression Profile Defines a Novel Glioblastoma Multiform (GBM) Gene-Mutation Signature. J Neurooncol (2019) 141(1):57–70. doi: 10.1007/s11060-018-03029-3 30414097

[6] Benayoun BA Pollina EA Ucar D Mahmoudi S Karra K Wong ED. H3K4me3 Breadth is Linked to Cell Identity and Transcriptional Consistency. Cell (2014) 158(3):673–88. doi: 10.1016/j.cell.2014.06.027 PMC4137894 25083876

[7] Chen H Li C Peng X Zhou Z Weinstein JN Liang H. A Pan-Cancer Analysis of Enhancer Expression in Nearly 9000 Patient Samples. Cell (2018) 173(2):386–399.e12. doi: 10.1016/j.cell.2018.03.027 29625054 PMC5890960

[8] Li T Fan J Wang B Traugh N Chen Q Liu JS Li B.. TIMER: A Web Server for Comprehensive Analysis of Tumor-Infiltrating Immune Cells. Cancer Res (2017) 77(21):e108–10. doi: 10.1158/0008-5472.CAN-17-0307 PMC6042652 29092952

[9] Bindea G Mlecnik B Tosolini M Kirilovsky A Waldner M Obenauf AC. Spatiotemporal Dynamics of Intratumoral Immune Cells Reveal the Immune Landscape in Human Cancer. Immunity (2013) 39(4):782–95. doi: 10.1016/j.immuni.2013.10.003 24138885

[10] Li JH Liu S Zhou H Qu LH Yang JH.. Starbase V2.0: Decoding miRNA-ceRNA, miRNA-ncRNA and Protein-RNA Interaction Networks From Large-Scale CLIP-Seq Data. Nucleic Acids Res (2014) 42(Database issue):D92–7. doi: 10.1093/nar/gkt1248 PMC3964941 24297251

[11] Qi X Zhang DH Wu N Xiao JH Wang X Ma W. ceRNA in Cancer: Possible Functions and Clinical Implications. J Med Genet (2015) 52(10):710–8. doi: 10.1136/jmedgenet-2015-103334 26358722

[12] Siegel RL Miller KD Fuchs HE Jemal A. Cancer Statistics, 2022. CA Cancer J Clin (2022) 72(1):7–33. doi: 10.3322/caac.21708 35020204

[13] Rajan P Stockley J Sudbery IM Fleming JT Hedley A Kalna G. Identification of a Candidate Prognostic Gene Signature by Transcriptome Analysis of Matched Pre- and Post-Treatment Prostatic Biopsies From Patients With Advanced Prostate Cancer. BMC Cancer (2014) 14:977. doi: 10.1186/1471-2407-14-977 25519703 PMC4301544

[14] Giotti B Chen SH Barnett MW Regan T Ly T Wiemann S. Assembly of a Parts List of the Human Mitotic Cell Cycle Machinery. J Mol Cell Biol (2019) 11(8):703–18. doi: 10.1093/jmcb/mjy063 PMC6788831 30452682

[15] Wang M Mao C Ouyang L Liu Y Lai W Liu N. Long Noncoding RNA LINC00336 Inhibits Ferroptosis in Lung Cancer by Functioning as a Competing Endogenous RNA. Cell Death Differ (2019) 26(11):2329–43. doi: 10.1038/s41418-019-0304-y PMC6889193 30787392

[16] Zhang L Hao C Zhai R Wang D Zhang J Bao L. Downregulation of Exosomal Let-7a-5p in Dust Exposed- Workers Contributes to Lung Cancer Development. Respir Res (2018) 19(1):235. doi: 10.1186/s12931-018-0949-y 30497474 PMC6267915

[17] Mu X Wu H Liu J Hu X Wu H Chen L. Long Noncoding RNA TMPO-AS1 Promotes Lung Adenocarcinoma Progression and Is Negatively Regulated by miR-383-5p. BioMed Pharmacother (2020) 125:109989. doi: 10.1016/j.biopha.2020.109989 32062549

