# Supplementary material for: Identifying Immune Cell Infiltration and Effective Diagnostic Biomarkers in Lung Adenocarcinoma by Comprehensive Bioinformatics Analysis and In Vitro Study
Source: Front Oncol. 2022 May 30;12:916947. doi: 10.3389/fonc.2022.916947 (PMC9189382; doi:10.3389/fonc.2022.916947)
Supplement: Supplementary file 1 [file Table_1.docx]

Supplementary Material

**Supplementary Table 1 miRNAs of FAM72B determined by starbase database.**

| miRNAid | miRNAname | miRNAid | miRNAname |  |
| --- | --- | --- | --- | --- |
| MIMAT0000062 | hsa-let-7a-5p | MIMAT0002883 | hsa-miR-514a-3p |  |
| MIMAT0000063 | hsa-let-7b-5p | MIMAT0003281 | hsa-miR-613 |  |
| MIMAT0000064 | hsa-let-7c-5p | MIMAT0003294 | hsa-miR-625-5p |  |
| MIMAT0000065 | hsa-let-7d-5p | MIMAT0003329 | hsa-miR-411-5p |  |
| MIMAT0000066 | hsa-let-7e-5p | MIMAT0003329 | hsa-miR-411-5p |  |
| MIMAT0000067 | hsa-let-7f-5p | MIMAT0003339 | hsa-miR-421 |  |
| MIMAT0000096 | hsa-miR-98-5p | MIMAT0004761 | hsa-miR-483-5p |  |
| MIMAT0000102 | hsa-miR-105-5p | MIMAT0004921 | hsa-miR-889-3p |  |
| MIMAT0000242 | hsa-miR-129-5p | MIMAT0004947 | hsa-miR-885-5p |  |
| MIMAT0000414 | hsa-let-7g-5p | MIMAT0004949 | hsa-miR-877-5p |  |
| MIMAT0000415 | hsa-let-7i-5p | MIMAT0004953 | hsa-miR-873-5p |  |
| MIMAT0000416 | hsa-miR-1-3p | MIMAT0004957 | hsa-miR-760 |  |
| MIMAT0000441 | hsa-miR-9-5p | MIMAT0005797 | hsa-miR-1301-3p |  |
| MIMAT0000455 | hsa-miR-185-5p | MIMAT0014983 | hsa-miR-3121-3p |  |
| MIMAT0000462 | hsa-miR-206 | MIMAT0019950 | hsa-miR-1245b-5p |  |
| MIMAT0000710 | hsa-miR-365a-3p | MIMAT0020541 | hsa-miR-5047 |  |
| MIMAT0000735 | hsa-miR-380-3p | MIMAT0022479 | hsa-miR-5688 |  |
| MIMAT0000737 | hsa-miR-382-5p | MIMAT0022701 | hsa-miR-506-5p |  |
| MIMAT0000773 | hsa-miR-346 | MIMAT0022724 | hsa-miR-1277-5p |  |
| MIMAT0002178 | hsa-miR-487a-3p | MIMAT0022834 | hsa-miR-365b-3p |  |
| MIMAT0002817 | hsa-miR-495-3p |  |  |  |
